# Poster Session II - A230 ULTRASOUND ELASTOGRAPHY IN INFLAMMATORY BOWEL DISEASE: A SYSTEMATIC REVIEW OF TECHNIQUES, PROTOCOLS, AND CLINICAL UTILITY TO DIFFERENTIATE FIBROSIS FROM INFLAMMATION

**DOI:** 10.1093/jcag/gwaf042.230

**Published:** 2026-02-13

**Authors:** R Khalaf, S Hoque, E Mak, M O’Brien, M Allocca, E Calabrese, K Gecse, G Maconi, A Medellin, K Novak, A Poulsen, R Panaccione, F Rieder, J St-Pierre, R Wilkens, S Wilson, S Sagami, C Lu

**Affiliations:** University of Calgary, Calgary, AB, Canada; University of Calgary, Calgary, AB, Canada; University of Calgary, Calgary, AB, Canada; University of Calgary, Calgary, AB, Canada; Universita Vita Salute San Raffaele, Milan, Lombardy, Italy; Universita degli Studi di Roma Tor Vergata, Rome, Lazio, Italy; Amsterdam Universitair Medische Centra, Amsterdam, NH, Netherlands; Universita degli Studi di Milano, Milan, Lombardy, Italy; University of Calgary, Calgary, AB, Canada; University of Calgary, Calgary, AB, Canada; Kobenhavns Universitet, Copenhagen, Capital Region of Denmark, Denmark; University of Calgary, Calgary, AB, Canada; Cleveland Clinic, Cleveland, OH; University of Calgary, Calgary, AB, Canada; Hvidovre Hospital, Hvidovre, Capital Region of Denmark, Denmark; University of Calgary, Calgary, AB, Canada; Kitasato University, Minato, Japan; Medicine, University of Calgary, Calgary, AB, Canada

## Abstract

**Background:**

Elastography has emerged as a promising adjunct to intestinal ultrasound (IUS) for assessing bowel stiffness and fibrosis in inflammatory bowel disease (IBD).Although elastography holds promise to differentiate fibrosis from inflammation in IBD, its clinical utility remains limited due to methodological challenges.

**Aims:**

We aim to summarize the technical performance and accuracy of elastography to identify fibrosis in both Crohn’s disease (CD) and ulcerative colitis (UC) on IUS in a systematic review.

**Methods:**

Systematic review using Embase, MEDLINE and Scopus databases was conducted up to August 19^th^ 2025. Risk of bias was graded using QUADAS-2. Data extracted included study design, patient demographics, (age, gender), fasting status, IUS machine model, probe type, elastography modality and technique, reference standard and reported outcomes. Bowel activity measurements such as thickness, Doppler signal, and stricture parameters were collected when available. To distinguish fibrosis from inflammation, only studies with comparison of elastography to a full thickness histologic gold standard were included.

**Results:**

Twenty-four studies, comprising 672 CD and 70 UC patients were included. Of the 24 studies, 17 (71%) studies (411 patients) included full-thickness histopathology and two (8%) studies (139 patients) included endoscopic biopsies. Nineteen (79%) studies were exclusively adults (535 patients), two (8%) studies were mixed adult-pediatric cohorts (136 patients) and three (13%) studies were pediatrics only (71 patients). Elastography protocols were highly heterogeneous, with 50% (12) studies (287 patients) utilizing strain elastography (SE), a qualitative measure of stiffness and 50% (12 studies) (400 patients) used shear wave elastography (SWE), a quantitative measure of stiffness. SWE has highest diagnostic accuracy to detect fibrosis in CD with sensitivity and specificity up to 75.0% and 100.0%, respectively when compared to SE. Two (8%) studies (60 patients) evaluated elastography in UC with full thickness resection samples. Across studies, SWE consistently showed higher stiffness values in fibrotic compared to inflammatory bowel segments, while SE distinguished patterns of inflammation and fibrosis, but with more variability.

**Conclusions:**

Elastography remains a promising tool for non-invasive fibrosis assessment in IBD. This systematic review highlights the need for standardized protocols, validated cutoff values, and multicenter studies comparing elastography to histological assessments using validated fibrosis indices to establish elastography as a routine clinical tool.

**Funding Agencies:**

None

